# Extracorporeal blood purification therapies for sepsis-associated acute kidney injury in critically ill patients: expert opinion from the SIAARTI-SIN joint commission

**DOI:** 10.1007/s40620-023-01637-5

**Published:** 2023-07-13

**Authors:** Silvia De Rosa, Marita Marengo, Marco Fiorentino, Vito Fanelli, Nicola Brienza, Enrico Fiaccadori, Giacomo Grasselli, Santo Morabito, Vincenzo Pota, Stefano Romagnoli, Fabrizio Valente, Vincenzo Cantaluppi, The SIAARTI-SIN joint commission

**Affiliations:** 1 Centre for Medical Sciences-CISMed, University of Trento, Trento, Italy; 2 Anesthesia and Intensive Care, Santa Chiara Regional Hospital, APSS Trento, Trento, Italy; 3 Nephrology and Dialysis Unit, Department of Specialist Medicine, Azienda Sanitaria Locale (ASL) CN1, Cuneo, Italy; 4 Nephrology, Dialysis and Transplantation Unit, Department of Precision and Regenerative Medicine and Ionian Area (DiMePRe-J), University of Bari, Bari, Italy; 5 Anesthesia, Critical Care and Emergency Unit, Department of Surgical Sciences, Città della Salute e della Scienza di Torino, University of Torino, Turin, Italy; 6 Unit of Anesthesia and Resuscitation, Department of Emergency and Organ Transplantations, University of Bari, Bari, Italy; 7 Dipartimento di Medicina e Chirurgia, UO Nefrologia, Azienda Ospedaliero-Universitaria Parma, Università di Parma, Parma, Italy; 8 Department of Anesthesia, Intensive Care and Emergency, Fondazione IRCCS Ca’Granda Ospedale Maggiore Policlinico, Milan, Italy; 9 Department of Pathophysiology and Transplantation, University of Milano, Milan, Italy; 10 UOSD Dialisi, Azienda Ospedaliero-Universitaria Policlinico Umberto I, “Sapienza” Università di Roma, Rome, Italy; 11 Department of Women, Child, General and Specialty Surgery, L. Vanvitelli University of Campania, Naples, Italy; 12 Department of Anesthesia and Critical Care, Azienda Ospedaliero-Universitaria Careggi, University of Firenze, Florence, Italy; 13 Nephrology and Dialysis Unit, Santa Chiara Hospital, APSS Trento, Trento, Italy; 14 Nephrology and Kidney Transplantation Unit, Department of Translational Medicine (DIMET), SCDU Nefrologia e Trapianto Renale, University of Piemonte Orientale (UPO), Azienda Ospedaliero-Universitaria Maggiore della Carità, via Solaroli 17, 28100 Novara, Italy

**Keywords:** Extracorporeal blood purification therapies, Sepsis-associated AKI, Cytokine removal, Endotoxin removal, Italian Society of Anaesthesia Analgesia Reanimation and Intensive Care, Italian Society of Nephrology

## Abstract

Sepsis-Associated Acute Kidney Injury is a life-threatening condition leading to high morbidity and mortality in critically ill patients admitted to the intensive care unit. Over the past decades, several extracorporeal blood purification therapies have been developed for both sepsis and sepsis-associated acute kidney injury management. Despite the widespread use of extracorporeal blood purification therapies in clinical practice, it is still unclear when to start this kind of treatment and how to define its efficacy. Indeed, several questions on sepsis-associated acute kidney injury and extracorporeal blood purification therapy still remain unresolved, including the indications and timing of renal replacement therapy in patients with septic vs. non-septic acute kidney injury, the optimal dialysis dose for renal replacement therapy modalities in sepsis-associated acute kidney injury patients, and the rationale for using extracorporeal blood purification therapies in septic patients without acute kidney injury. Moreover, the development of novel extracorporeal blood purification therapies, including those based on the use of adsorption devices, raised the attention of the scientific community both on the clearance of specific mediators released by microorganisms and by injured cells and potentially involved in the pathogenic mechanisms of organ dysfunction including sepsis-associated acute kidney injury, and on antibiotic removal. Based on these considerations, the joint commission of the Italian Society of Anesthesiology and Critical Care (SIAARTI) and the Italian Society of Nephrology (SIN) herein addressed some of these issues, proposed some recommendations for clinical practice and developed a common framework for future clinical research in this field.

**Graphical abstract:**

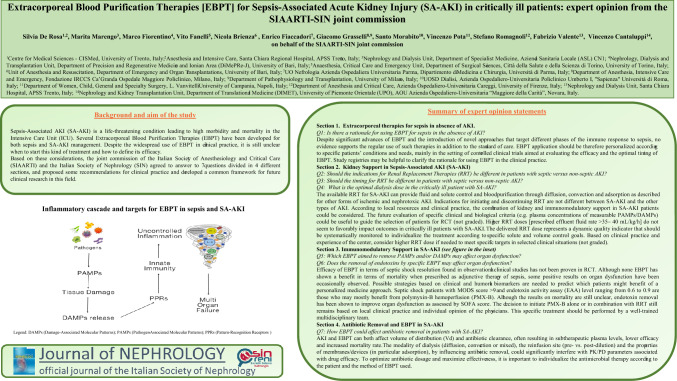

## Introduction

Sepsis accounts for about 50% of all patients with acute kidney injury (AKI) and represents the leading cause of death in the Intensive Care Unit (ICU) [1]. Sepsis-Associated AKI (SA-AKI) is a life-threatening complication leading to high morbidity and mortality in critically ill patients [2]. Many aspects of SA-AKI are poorly described including clinical definition, epidemiology, pathogenic mechanism, impact of resuscitative and fluid strategies, role of biomarkers for risk stratification, diagnosis, treatment guidance and potential impact on short- and long-term outcomes [3]. Of note, molecules released by different microorganisms (Pathogen Associated Molecular Patterns [PAMPs]) or by injured cells (Damage Associated Molecular Patterns [DAMPs]) into the bloodstream have been shown to be deeply involved in the mechanisms of SA-AKI, thus becoming an attractive therapeutic target.

During the past decades, several Extracorporeal Blood Purification Therapies (EBPTs) have been developed for both sepsis and SA-AKI management. However, the lack of established guidelines has led to a high degree of variability in clinical practice: for this reason, it is presently uncertain what the optimal conditions for initiation and discontinuation of EBPTs are, and how to define treatment efficacy. The inconclusive results of Randomized Clinical Trials (RCTs) may be mainly related to the heterogeneity of septic patients included in the studies: SA-AKI should therefore be considered a syndrome in which various factors can contribute to tissue damage, with an urgent need for future research to identify a specific subphenotype of patients who may potentially benefit from EBPT.

For all these reasons, the Italian Society of Anesthesiology and Critical Care (SIAARTI) and the Italian Society of Nephrology (SIN) joint commission herein aimed to address the above-mentioned issues, to propose some recommendations for clinical practice, and to develop a common framework for further clinical and translational research in this field. Thus, the SIAARTI-SIN expert panel identified seven of the most relevant questions in this specific clinical context that are discussed in the following four sections of the present manuscript.

## Section 1. Extracorporeal therapies for sepsis in the absence of AKI

*Q1: Is there a rationale for using EBPT for sepsis in the absence of AKI?*

Consensus statements:

Despite significant advances of EBPT and the introduction of novel approaches targeting different phases of the immune response to sepsis, no evidence supports the regular use of such therapies in addition to the standard of care.

Extracorporeal blood purification therapies should therefore be personalized according to the patients’ specific conditions and needs, mainly in the setting of controlled clinical trials aimed at evaluating the efficacy and the optimal timing of EBPT.

Study registries may be helpful to clarify the clinical results and the rationale for using EBPT in clinical practice.

### Rationale

**Fig. 1:**
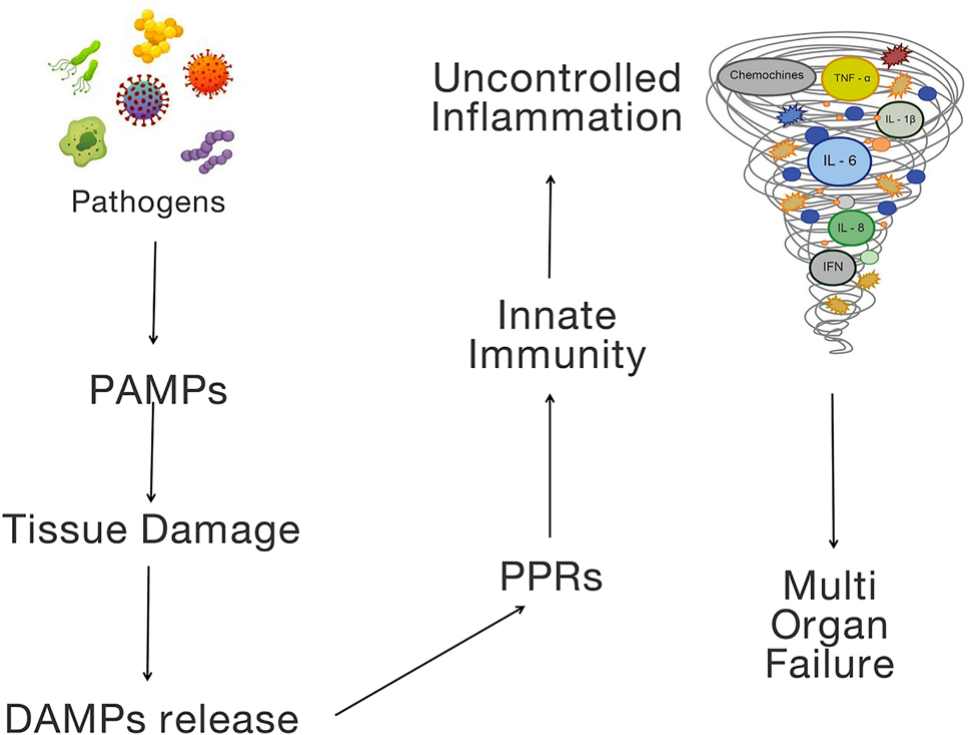
Inflammatory cascade and targets for extracorporeal blood purification therapies [EBPT] in sepsis. *DAMPs* damage-associated molecular patterns, *PAMPs* pathogen-associated molecular patterns, *PPRs* pattern-recognition receptors

Despite recent advances in diagnosis and management, sepsis still represents a major global health concern, with rising incidence and mortality [2]. Adequate source control and appropriate antibiotic therapy are well-recognized strategies to reduce the impact of this syndrome; however, the increasingly observed antibiotic resistance and the absence of specific therapies limit the possibility to treat sepsis and septic shock [4]. Novel treatment strategies have been proposed in the last few years (including early and aggressive fluid resuscitation strategies, vasopressors/inotropic support and multi-organ support) however no improvement in clinical outcomes has been observed. In this clinical scenario, EBPTs have been suggested for treating sepsis with and without renal dysfunction, though with controversial results [5]. The rationale for their use in clinical practice is related to the pathogenesis of sepsis and sepsis-associated organ dysfunction. Sepsis is currently defined as a life-threatening organ dysfunction caused by a dysregulated host response to infection with a consequent imbalance between the pro- and the anti-inflammatory response [6]. The first step of the host immune response is the recognition of specific molecules expressed by PAMPs by specific receptors expressed by different cell types (Toll-like receptors [TLRs]), thus activating the innate immunity and leukocytes and the release of pro- and anti-inflammatory cytokines [cytokine storm], leading to a dysregulated host response and multiple organ dysfunction [7]. Similarly, a vicious cycle takes place, since the damaged host cells release specific proteins, i.e., DAMPs, that can amplify this dysregulated response and increase the risk of multi-organ failure [8]. In this setting, EBPTs may potentially help in removing specific triggers and patterns involved in the inflammatory cascade, inducing immunomodulation and potentially contributing to organ protection (Fig. 1).

To date, several EBPTs for immunomodulatory support have been proposed in clinical practice aimed at targeting the immune response at different stages. Some EBPTs act by removing PAMPs (e.g. endotoxins) from the blood using hemoperfusion techniques with specific sorbents such as Polymyxin-B hemoperfusion PMX-HA (Polymyxin-B Hemoadsorption), now called PMX-HA (Polymyxin-B Hemoadsorption) and the adsorber characterized by a tailor-made peptide designed to bind the Lipid-A, the toxic part of endotoxin and Gram negative bacteria. In addition, several adsorption devices have been introduced in clinical practice for the removal of cytokines: these sorbents may be used alone (direct hemoperfusion or plasma filtration and adsorption) or in series with a standard hemodialysis membrane when necessary because of the presence of AKI. Cytokine adsorption cartridges made of polystyrene divinylbenzene copolymer beads with a molecular cut-off size of 60 kDa, are widely used to allow the adsorption of both pro- and anti-inflammatory mediators, myoglobin, bilirubin, DAMPs and PAMPs except for endotoxins [8]. In addition, strategies aimed at targeting activated leukocytes have been tested in experimental studies and in clinical trials (Selective Cytopheretic Device) [9]. Finally, a novel technique based on the use of a sorbent that directly removes a wide range of pathogens from the blood has been proposed as adjuvant therapy during bloodstream infection with promising in vitro results. In this setting, various hypotheses have been considered to support the importance of blood purification in sepsis and septic shock [10]. Ronco et al. proposed the peak concentration hypothesis, according to which the production of pro- and anti-inflammatory cytokines may occur at different time-points and a non-selective control of the peaks of soluble inflammatory mediators may be effective in reducing the degree of imbalance and improving immune system homeostasis [11]. Moreover, the cytokinetic theory supports the hypothesis that the removal of inflammatory mediators from the plasma may create a gradient between plasma and tissues, favoring the migration of detrimental substances into the bloodstream, thus preventing organ dysfunction [12]. However, although some pathogenetic mechanisms of sepsis could support the use of EBPT even in the absence of renal dysfunction, the potential adverse effects of these techniques should be taken into consideration: the need for a central catheter and the increased risk of thrombosis and severe infections, anticoagulation to prevent circuit clotting and the resulting increased risk of bleeding, the potential loss of nutrients and electrolytes [e.g. hypophosphatemia] and antibiotic removal with consequent underdosing [13]. These considerations represent a limit for the widespread use of these devices, supporting the idea of a personalized use of EBPT according to the patients’ specific needs and after weighing risks and benefits from such treatments: presently, the use of EBPT should be mainly recommended within RCTs and/or in study registries.

## Section 2. Kidney support in sepsis-associated AKI

*Q2: Should the indications for Renal Replacement Therapies be different in patients with septic versus non-septic AKI?*

*Q3: Should the timing of RRT be different in patients with septic versus non-septic AKI?*

Consensus statements:

The available RRT for SA-AKI can provide fluid and solute control and blood purification through diffusion, convection and adsorption as described for other forms of ischemic and nephrotoxic AKI.

Indications for initiating and discontinuing RRT do not differ between SA-AKI and other types of AKI.

Depending on local resources and clinical practice, the combination of kidney and immunomodulatory support in SA-AKI patients could be considered. Future evaluation of specific clinical and biological criteria (e.g. plasma concentrations of measurable PAMPs/DAMPs) could be useful to guide the selection of patients for RCTs (not graded).

### Rationale

Currently, sepsis represents the main cause of AKI in the ICU, and the concomitant presence of sepsis and AKI worsens outcome, in particular for patients requiring RRT. In a cohort of patients with sepsis included in the Genetic and Inflammatory Markers of Sepsis (GenIMS) study, long-term survival was strongly influenced by renal recovery [13]. For these reasons, recent studies explored the potential differences between SA-AKI and other forms of AKI for RRT indications. Moreover, the mechanisms of SA-AKI have recently been better elucidated, showing that tissue damage is not merely ascribed to hypoperfusion, but to causes that are more toxic and immunologic in nature, including the presence in the bloodstream of PAMPs [e.g. LPS] and DAMPs [e.g. HMGB-1] able to contribute to organ dysfunction including AKI [7, 14, 15]. When we consider the standard indications to start RRT, some considerations are valid for SA-AKI as well as for all other causes of renal dysfunction: the Kidney Disease: Improving Global Outcomes (KDIGO) 2012 guidelines recommend initiating RRT in the presence of life-threatening complications related to AKI such as electrolyte/acid–base alterations and fluid overload, or for conditions that can be modified by RRT. Several clinical trials confirmed that fluid overload has become the most relevant indication for RRT start, mostly in the presence of sepsis: moreover, fluid overload is associated with organ dysfunction and increased mortality in AKI patients, suggesting that an earlier RRT start might be beneficial [15, 16]. Based on this consideration, in the last years several RCTs compared early vs. late RRT start, though with controversial results. In 2016, the single-center Early versus late initiation of renal replacement therapy in critically ill patients with acute kidney injury (ELAIN) study including 231 critically ill patients with AKI showed that early RRT [within 8 h of diagnosis of KDIGO stage 2] reduced mortality over the first 90 days [17]. In the same year, the Artificial Kidney Initiation in Kidney Injury (AKIKI) trial, an open label 31-center study including 620 patients in French ICUs, revealed no significant difference in mortality between an early vs. a delayed strategy of RRT initiation: in addition, the delayed strategy averted the need for RRT in a considerable number of patients [18]. Afterward, the Initiation of Dialysis Early Versus Delayed in the Intensive Care Unit (IDEAL-ICU) study, a multicenter trial with inclusion criteria similar to AKIKI and recruiting patients with severe AKI [KDIGO stage 3] in addition to early-stage septic shock [within 48 h from vasopressor initiation], showed a similar 90-day mortality in the early vs. delayed modality, without investigating fluid balance between the 2 groups [19]. A more recent RCT showed that earlier RRT start did not confer a survival advantage, by contrast increasing the risk of harm: the Standard versus Accelerated Initiation of Renal Replacement Therapy in Acute Kidney Injury (STARRT-AKI) trial randomly assigned patients with severe AKI to receive an accelerated [12 h from eligibility criteria] vs. a standard RRT strategy, without finding any significant difference in 90-day mortality between the 2 groups [20]. The AKIKI-2 was a multicenter RCT conducted in 39 French ICUs demonstrating that with respect to the delayed strategy of AKIKI and in the absence of severe AKI-related complications, a longer RRT delay did not confer additional benefit, on the contrary leading to potential harm [21]. Taken together, all the reported RCTs lead to the conclusion that in the presence of different forms of AKI including SA-AKI, RRT should be started neither too early nor too late for a specific patient in accordance with a “personalized medicine” approach.

A similar controversial condition is also observed when defining the need for RRT discontinuation: in this setting, prolonged dialysis duration might be associated with a reduced chance of regaining kidney function. In a recent systematic review and meta-analysis aimed at determining the optimal time for RRT interruption (DOnE RRT), the increase in urine output and the consequent resolution of fluid overload were the most often described and robust predictors [22].

A recent meta-analysis showed that blood NGAL/cystatin C as well as urinary TIMP-2/IGFBP-7 are the best predictors for RRT initiation. However, the current strength of evidence excludes the routine use of biomarkers to guide the decision-making for starting [and stopping] RRT, with the need for more research on this topic [23]. The use of biomarkers may improve the accuracy of different “AKI bundles” leading to earlier recognition and management of patients requiring RRT.

As described above, many studies have investigated the role of different EBPTs to remove PAMPs and DAMPs from the bloodstream in the course of SA-AKI [6, 13, 14]. These harmful mediators are known to induce a series of sub-lethal and lethal alterations of kidney endothelial cells and tubular epithelial cells including loss of polarity, inflammation, bioenergetic derangement due to mitochondrial dysfunction, senescence and apoptotic cell death [24–26]. On this basis, the use of membranes and/or devices with enhanced permeability or adsorptive properties (direct hemoperfusion or plasma filtration and adsorption) has been proposed even independently from the standard indications for RRT and with a possible different timing. Despite these potential protective effects, current guidelines do not support the use of EBPT to improve patient outcomes [27]. Further research is needed to determine the effects of EBPT in real-life ICU settings, focusing on different clinical endpoints not only related to hard outcomes (mortality), but also to early- and long-term evaluation of organ function and immune response in survivors.

*Q4: What is the optimal dialysis dose in the critically ill patient with SA-AKI?*

Consensus statements:

Higher RRT doses [prescribed effluent fluid rate > 35–40 mL/kg/h] do not seem to favorably impact outcomes in critically ill patients with SA-AKI.

The delivered RRT dose represents a dynamic quality indicator that should be systematically monitored to individualize the treatment according to specific solute and volume control goals.

Based on clinical practice and experience of the center, consider higher RRT dose if needed to meet specific targets in selected clinical situations (not graded).

### Rationale

Several RCTs have been performed with the aim to assess the optimal dose of RRT: the provision of RRT in AKI patients requires timely prescription and a specific dose in order to achieve adequate solute removal and volume control [28]. The total effluent fluid rate is the preferred parameter used to assess RRT dose according to different modalities (Table 1). There have been several interventional studies examining the relationship between RRT dose and overall survival or kidney recovery. Controversial results are reported in RCTs that compared the effect of dialytic dose in different RRT modalities. In the landmark “Vicenza study”, Ronco et al. showed a significantly greater survival rate in patients who received a prescribed dialytic dose of 35 or 45 ml/kg/h compared to those with a dialytic dose of 20 mg/kg/h in post-dilution continuous venovenous hemofiltration (CVVH) [29]. Saudan et al. [30] reported a significant increase in 90-day survival in the higher-intensity treatment group (34% in the CVVH group vs 59% in the continuous venovenous hemodiafiltration [CVVHDF] group). Conversely, two other studies failed to demonstrate any significant difference in mortality or kidney recovery when higher-intensity therapies (CVVH or CVVHDF at 35–45 ml/kg/h) were compared to the standard dose (20 ml/kg/h) [31, 32]. Similarly, in the Dobutamine Compared with Milrinone (DO-RE-MI) study, RRT dose higher than 35 mL/kg/h was not associated with increased survival even after adjustment for baseline characteristics [33]. Based on the discrepancies showed by these small studies, two larger multi-center RCTs were conducted; the US Acute Renal Trial Network (ATN) trial and the Australia/New Zealand Randomized Evaluation of Normal versus Augmented Level (RENAL) trial. In the ATN study, 1124 patients were randomized to receive pre-dilution CVVHDF with a total effluent flow rate of 35 ml/kg/h or 6 sessions/week of intermittent dialysis vs. pre-dilution CVVHDF at 20 ml/kg/h or 3 sessions/week of intermittent treatments; 60-day mortality was similar between the groups [46% vs 48%] and no differences in kidney recovery were documented [34]. Similarly, the RENAL study, that randomized 1508 critically ill patients to receive post-dilution CVVHDF with an effluent dose of 25 vs 40 ml/kg/h, failed to report any significant effect on mortality and kidney recovery [35]. Finally, a 2016 Cochrane systematic review including 6 studies with a total of 3185 participants did not demonstrate improved mortality or kidney recovery rate with a more intensive RRT approach, except for a subgroup of post-surgical AKI patients; furthermore, an increased risk of hypophosphatemia was documented [36]. The hypothesis of the potential benefit of a more intensive RRT dose in specific subgroups of patients has been investigated: Clark et al. performed a systematic review and meta-analysis of 4 RCTs (including the most recent High-volume versus standard-volume hemofiltration for septic shock patients with acute kidney injury [IVOIRE] study) investigating the benefits of high-volume hemofiltration compared to standard volume hemofiltration in the treatment of sepsis and septic shock. None of the considered RCTs showed any improvement in 28-day mortality or in any secondary outcomes (kidney recovery, length of ICU stay, vasopressor use) [37].

**Table 1:** Determination of total effluent rate among RRT modalities

CVVH
Total effluent rate = total UF rate (sum of pre-filter and post-filter replacement fluid rate, ml/h) + fluid removal rate (ml/h)
CVVHD
Total effluent rate = dialysate rate (ml/h) + fluid removal rate (ml/h)
CVVHDF
Total effluent rate = total UF rate (sum of pre-filter and post-filter replacement fluid rate, ml/h) + dialysate rate (ml/h) + fluid removal rate (ml/h)
Dilution factor for pre-dilution
Plasma flow rate (ml/h)/[plasma flow rate (ml/h) + pre-filter replacement fluid rate (ml/h)]
Plasma flow rate (ml/h) = blood flow rate (ml/min) × 60 (min/h) x (1 – HCT)

*RRT* renal replacement therapy, *CVVH* continuous venovenous hemofiltration, *CVVHD* continuous venovenous hemodialysis, *CVVHDF* continuous venovenous hemodiafiltration, *UF* ultrafiltration, *HCT* current patient hematocrit

Based on this evidence, the KDIGO AKI guidelines recommend delivering an average effluent dose of 20–25 mL/kg/h for patients with AKI requiring RRT. Noteworthy, the prescribed RRT dose is not always delivered as several specific factors (interruptions related to radiologic or surgical procedures, circuit downtime due to clotting/clogging, replacing filters, bag/tubing changes, dialysis catheter issues, etc.) may influence the delivered RRT dose. Therefore, periodic evaluation of the delivered dose and solute/volume control goals is strongly suggested to adjust RRT prescription and to tailor the treatment and dose according to the patient’s needs using a personalized medicine approach. No proven strategies have been reported to compensate a decreased delivered dose due to circuit downtime. Accounting for an average 10–15% of circuit downtime during RRT, the total prescribed effluent dose should be at least 25–30 mL/kg/h (10–15% above the recommended effluent dose) [38].

In summary, there is no evidence to support that a higher RRT dose (prescribed effluent fluid rate > 35– 40 mL/kg/h) favorably impacts outcomes in critically ill patients with AKI when compared to a standard RRT dose (prescribed effluent fluid rate 25–30 mL/kg/h), even in case of SA-AKI. The delivered dose represents a dynamic quality indicator that should be systematically monitored to individualize the treatment according to specific solute or volume control goals.

## Section 3. Immunomodulatory support in SA-AKI

*Q5: Which EBPTs aimed at removing PAMPs and/or DAMPs could affect organ dysfunction?*

Consensus statements:

The efficacy of EBPTs in terms of septic shock resolution found in observational clinical studies has not been proven in RCTs.

Although none of the EBPTs has shown a benefit in terms of mortality when prescribed as adjuvant therapy of sepsis, some positive results on organ dysfunction have occasionally been observed.

Possible strategies based on clinical and humoral biomarkers are needed to predict which patients might benefit from a personalized medicine approach.

### Rationale

As previously reported, pro- and anti-inflammatory cytokines are released into the bloodstream by several cell types in response to PAMPs and DAMPs, key mediators of innate and adaptive immune systems able to initiate and downregulate immune response to re-establish homeostasis. However, the “cytokine storm” characterized by overwhelming release of these mediators is associated with the risk of multi-organ dysfunction syndrome (MODS) and death in severely ill patients [39]. New technological advances in EBPTs prompted the idea of mitigating cytokine overproduction and life-threatening hyper-inflammation by three main mechanisms: 1. High volume hemofiltration: RRT with high convective target dose (> 35 ml/kg/h); 2. High cut-off membranes: use of membranes with large pore size (average diameter 20 nm); 3. Adsorption: use of RRT membranes with adsorptive properties or hemoperfusion/plasma filtration and adsorption devices combined or not with RRT. To date, none of these techniques has shown a clear benefit in terms of mortality when prescribed as adjuvant therapy for sepsis, uncontrolled inflammatory response to cardiopulmonary by-pass in cardiac surgery or in the case of COVID-19-associated pneumonia [40]. The conclusions of multiple case series and single center observational studies are hindered by small sample size: for this reason, we focused on findings reported in larger RCTs. In a matched cohort study, patients with refractory septic shock as defined by vasopressor dependency index > 3 despite adequate volume resuscitation and a value of IL-6 > 1000 ng/l were treated with a cytokine absorption device for 3 consecutive sessions starting within 24 h from the onset of septic shock. Over time, blood IL-6 concentrations and vasopressor requirements decreased both in treated patients and in the control group. However, ICU mortality was higher in patients subjected to cytokine absorption (67 vs. 42%) [41]. These findings have been confirmed in an open RCT of septic shock patients and acute respiratory distress syndrome who received daily hemoperfusion with the cytokine adsorber device 6 h per day for up to 7 days. Plasma levels of several cytokines and chemokines (IL-10, MCP-1, MIP-1 alpha, IL-1ra, IL-18 and VEGF), including IL-6 (elimination rate 5–18%), did not differ between the two groups. No differences were detected in time spent on mechanical ventilation and degree of multiorgan dysfunction. After adjusting for the proportion of patients under RRT and severity, mortality at 60 days was similar in both groups [41].

Cytokine adsorption has been advocated as part of immunomodulation therapy for COVID-19 patients with pneumonia and severe cytokine release syndrome. In an open label RCT, 17 patients with COVID-19 requiring ECMO were treated with a cytokine adsorber device that was replaced every 24 h, and removed after 72 h, vs 17 patients treated without a cytokine adsorber device. Clearance of IL-6 did not significantly differ between the two groups and crude mortality at 30 days was higher in patients who received cytokine adsorption [42]. Similar negative results on cytokine adsorption in septic patients were also observed using Coupled Plasma Filtration Adsorption in the COMbining Plasma-filtration and Adsorption Clinical Trial-2 (COMPACT-2) study that was prematurely stopped due to the increased mortality rate in the treated group [43].

Based on these results, current evidence does not support the routine use of EBPTs in SA-AKI. Further studies are needed to establish their clinical efficacy, in particular enrichment strategies based on biomarkers readily available at bedside to predict which patients may most benefit.

*Q6: Could the removal of endotoxins by specific EBPTs affect organ dysfunction?*

Consensus statements:

Septic shock patients with a MODS score > 9 and endotoxin activity assay (EAA) level ranging from 0.6 to 0.9 are those who may mostly benefit from polymyxin-B hemoperfusion.

Although the results on mortality are still unclear, endotoxin removal has been shown to improve organ dysfunction as assessed by the Sequential Organ Failure Assessment (SOFA) score.

The decision to initiate PMX-B alone or in combination with RRT still remains based on local clinical practice and individual opinion of the physicians. This specific treatment should be performed by a well-trained multidisciplinary team.

### Rationale

Endotoxin is one of the most harmful PAMPs identified in patients with septic shock [44]. Extracorporeal endotoxin neutralization has been extensively studied, in particular the therapy based on polymyxin B-immobilized polystyrene-derived fiber hemoperfusion conceived for direct endotoxin removal from whole blood. Although there is no definitive evidence supporting the efficacy of PMX‐B in a selected population with endotoxic shock, its current use has been challenged by a series of clinical trials [45–50] that showed no clear evidence to support its routine use to treat patients with septic shock. Two recent meta-analyses demonstrated a favorable effect on mortality using this EBPT modality [51, 52], although with controversial data analyses and results.

Zhou et al. conducted a systematic review and meta-analysis of RCTs to demonstrate the association between several EBPTs and overall mortality: pooling of all trials was not associated with a lower mortality rate when studies based on PMX-B performed in Japan were excluded [51]. Another meta-analysis revealed that PMX-B treatment may reduce mortality in patients with septic shock; in addition, the disease severity subgroup meta-analysis indicated a survival benefit related to PMX-B treatment in the intermediate- and high-risk groups. In the Evaluating the Use of Polymyxin B Hemoperfusion in a Randomized controlled trial of Adults Treated for Endotoxemia and Septic shock (EUPHRATES) RCT, patients with septic shock and EAA level higher than 0.6 received two PMX-B hemoperfusions. Mortality at 28 days did not differ between all patients and the critically ill with a MODS score higher than 9 [45]. A post-hoc analysis performed in 194 patients with an EAA between 0.6 and 0.89 showed improvement in mortality, ventilation-free days and median arterial pressure [46].

**Fig. 2:**
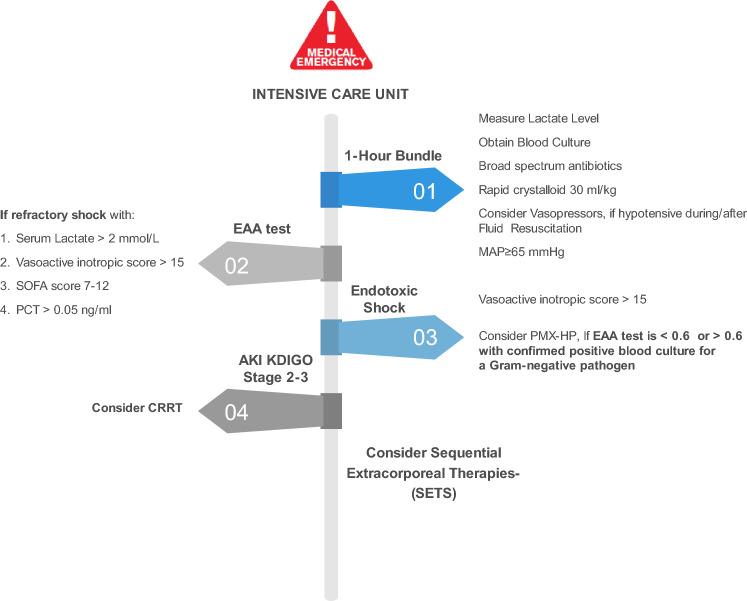
Approach to endotoxic shock

A golden hour for considering targeted use of PMX-B hemoperfusion as adjuvant therapy based on diagnosis and management of endotoxic shock has recently been suggested. This approach also includes the use of EAA evaluation at regular intervals, close to the source control, microbiological cultures and antibiotic administration [53] (Fig. 2). However, the evidence supporting this approach is insufficient.

When organ failure develops, sequential extracorporeal therapies may support or replace the function of different organs such as heart, kidney, liver and lung. However, although the Surviving Sepsis Campaign (SSC) guidelines 2016 did not make recommendations regarding the use of EBPT, the more recent 2021 SSC guidelines proposed a weak recommendation against the use of PMX-B and insufficient evidence to make recommendations for other EBPTs considering the resources required, costs and health equity issues [54].

Nowadays, the decision to initiate PMX-B alone or in combination with RRT is still based on local clinical practice and individual opinion of the physicians. Despite multiple studies, the optimal timing, modality, duration and anticoagulation strategies are still largely unclear.

## Section 4. Antibiotic removal and EBPT in SA-AKI

*Q7: How can EBPT affect antibiotic removal in patients with SA-AKI?*

Consensus statements:

AKI and EBPT can both affect volume of distribution and antibiotic clearance, often resulting in subtherapeutic plasma levels, lower efficacy and increased mortality rate.

The modality of dialysis (diffusion, convection or mixed), the reinfusion site (pre- vs. post-dilution) and the properties of membranes/devices (in particular adsorption) could significantly interfere with pharmacokinetic/pharmacodynamic parameters associated with drug efficacy by influencing antibiotic removal.

To optimize antibiotic dosage and maximize effectiveness, it is important to individualize the antimicrobial therapy based on the patient and the EBPT used.

### Rationale

Sepsis therapy is primarily based on source control and on organ function support. In this setting, antimicrobial therapy should be targeted as soon as the pathogen is identified, and adequate antibiotic dosing is essential to improve morbidity and mortality, particularly in SA-AKI patients. Choosing the appropriate antimicrobial regimen in SA-AKI can be difficult as many factors (clinical context, source of infection, AKI stage, etc.) must be taken into consideration and, to date, there are no validated guidelines on antibiotic dose adjustments in these patients.

The 2016 SSC Guidelines strongly recommend that dosing strategies of antimicrobials should be optimized based on pharmacokinetic and pharmacodynamic properties [55]. Pharmacokinetics is defined by adsorption, distribution, metabolism and elimination of a drug, whereas pharmacodynamics describes the impact of serum levels and drug response. Thus, the pharmacodynamics of an antimicrobial may be time-dependent, or concentration-dependent or both. To optimize and maximize antimicrobial therapy, as well as to reduce the risk of antimicrobial resistance and toxicity, the right drug should be selected and given at an appropriate dose based on its pharmacokinetic and pharmacodynamic properties [56]. Moreover, volume of distribution is one of the most important pharmacokinetic elements, usually modified during sepsis and AKI. Sepsis and early fluid resuscitation can induce pathophysiological changes such as altered fluid balance, hypoalbuminemia, capillary leakage, and kidney and liver dysfunction: all these variables can modify the pharmacokinetics of antimicrobial agents as well as of other drugs commonly used in critically ill patients [4]. Antibiotic doses are calculated based on preferred peak concentration and volume of distribution: higher doses are usually required during sepsis in order to achieve target antimicrobial concentrations [57]. Furthermore, AKI and EBPT can both affect volume of distribution and antibiotic clearance, often resulting in subtherapeutic plasma levels, lower efficacy and increased mortality rate [58]. Data on drug clearance to guide antimicrobial dosing in SA-AKI patients are limited and becoming outdated with the advances in technology for EBPT modality and efficiency. In addition, renal dosing recommendations are usually based on pharmacokinetic studies performed in patients with chronic kidney disease and in non–critically ill patients receiving scheduled intermittent hemodialysis; these recommendations are not appropriate in the context of AKI when glomerular filtration rate assessment is particularly inaccurate and RRT/EBPT modalities can vary daily [59, 60].

Therefore, in patients with SA-AKI, besides the pharmacokinetic features of the antibiotic (molecular weight, hydrophilicity, electric charge, protein binding and volume of distribution), standard RRT and EBPT modalities, setting and filter membrane types should be considered to assess the most appropriate antimicrobial dose.

The 3 main modalities of EBPT are based on convective, diffusive and mixed (both convective and diffusive) purification techniques. Antibiotic clearance during convective modalities is directly proportional to Sieving Coefficient (SC) and ultrafiltration rate, although they may be affected by the modality of reinfusion [pre- or post-dilution]. Conversely, antibiotic clearance is more challenging in the setting of diffusive and/or mixed modalities due to the large variability of saturation coefficient and it is proportional to effluent flow rate. Thus, high-intensity RRT may strongly impact antimicrobial clearance; higher/full doses and/or prolonged infusion of antibiotics should be considered when an effluent flow rate ≥ 3 l/h or post-dilution mode are used. On the other hand, a dose reduction should be considered for lower effluent rate or pre-dilution mode [61].

The choice of the dialyzer could also affect antibiotic clearance: high-flux membranes, with increased permeability to medium size molecules, have a greater capacity to remove drugs with a high molecular weight compared to low-flux membranes. Likewise, several membranes [polysulfone, polymethylmethacrylate, polyacrylonitrile, etc.], differ from each other according to their adsorptive ability and surface area and this could significantly interfere with antibiotic removal.

The combination of selective or non-selective adsorptive devices with standard RRT may further complicate the determination of the right antibiotic dose: studies aimed at evaluating antimicrobial adsorption by these devices are strongly recommended [62].

To optimize antibiotic dosage and maximize effectiveness, it is important to individualize the antimicrobial therapy to the patient and the method of RRT/EBPT utilized. This may be challenging for clinicians because it requires optimal knowledge of the different EBPT modalities and their effects on drug clearance as well as the role of sepsis on the pharmacokinetics/pharmacodynamics of the antibiotic. Lack of standardization of EBPT and RRT including fluid removal, membrane characteristics, duration of procedure, delivered dose and effluent rates have led to variability in published recommendations and, consequently, to the difficult and heterogeneous management of these patients [63–66].

## Conclusions

SA-AKI is best defined as the occurrence of AKI within 7 days of sepsis development (diagnosed according to KDIGO criteria and Sepsis 3 criteria, respectively). The identification of distinct endotypes of SA-AKI may provide crucial prognostic information as recently defined by the consensus report of the 28th Acute Disease Quality Initiative workgroup [67].

The indications to start RRT in SA-AKI do not differ from other causes of renal dysfunction. EBPT techniques might be considered for immunomodulatory support in patients who meet explicit clinical and biological criteria based on the measurement of specific detrimental molecules.

## Data Availability

No new data was generated for this report.
